# The fractional-order Lorenz-type systems: A review

**DOI:** 10.1007/s13540-022-00016-4

**Published:** 2022-04-18

**Authors:** Ivo Petráš

**Affiliations:** Faculty of BERG, Technical University of Košice, Němcovej 3, 042 00 Kosice, Slovak Republic

**Keywords:** Fractional calculus (primary), Fractional-order chaotic system, Chaotic oscillator, Attractor, 26A33 (primary), 34C28, 34A08

## Abstract

This paper deals with a survey of Lorenz-type systems. For the first time, a new classification of the fractional-order Lorenz-type systems was introduced. Several chaotic systems, as particular cases of the new general form, which belong to large Lorenz family, are presented together with equilibria, eigenvalues as well as attractors of these systems in 3-dimensional state space, respectively.

## Introduction

It is well-known that chaos theory concerns complex deterministic systems behaviour. This behaviour is known as a deterministic chaos and its investigation attracted many researcher during last few decades. Chaotic behaviour exists in many natural systems and disciplines, as for instance, meteorology, sociology, economics, engineering, ecology, chemistry, medicine, including covid-19 pandemic crisis management, and so on. One of the most popular chaotic system is a Lorenz oscillator.

The Lorenz oscillator is a 3-dimensional dynamical system that exhibits chaotic flow [11]. The Lorenz attractor was named according to Edward Norton Lorenz, who derived it from the simplified equations of convection rolls arising in the equations of the atmosphere in 1963. He for the first time used the term “butterfly effect" in his lecture named “Predictability: Does the Flap of a Butterfly’s Wings in Brazil Set Off a Tornado in Texas?” presented on December 29, 1972, at the conference of the American Association for the Advancement of Science. In chaos theory it means sensitive dependence on initial conditions and parameters. Small variations of initial condition or parameters of a dynamical system may produce large variations in the long term behaviour of the system. The phrase refers to the idea that a butterfly’s wings might create tiny changes in the atmosphere that may ultimately alter the path of a tornado or delay, accelerate or even prevent the occurrence of a tornado in a certain location. The flapping wing represents a small change in the initial condition of the system, which causes a chain of events leading to large-scale alterations of events.

Such behaviour of the chaotic system can be studied through the analysis of its mathematical model, as well as its graphical outputs as strange attractors, bifurcations, and Poincaré maps. The mathematical model is usually in the form of a set of ordinary differential equations or even more complex form, a set of fractional differential equations, where a fractional dynamics is incorporated.

In this article we will focus on generalized Lorenz-like systems described by a set of the fractional differential equations. Various known chaotic systems belong to this special class of the chaotic systems. Here, we present seven popular chaotic systems of this large family with butterfly wings-like attractors.

This paper is organized as follows. Section 1 introduced the problem. In Section 2 some preliminaries, as fractional calculus, fractional-order system model and its numerical solution are presented. Section 3 presents a survey of fractional-order Lorenz-type systems together with illustrative examples. In Section 4 some concluding remarks and further research ideas are discussed.

## Preliminaries

### Definition of fractional operator

The history of fractional calculus begun in letter from Leibniz to l’Hopital dated on September 30, 1695, where derivative of the order 1/2 was mentioned.

The fractional calculus is a generalization of integration and differentiation to joint non-integer *q*-order operator $_{a}D^{q}_{b}$, where *a* and *b* are the bounds of the operation. The standard notation for denoting the common left-sided integro-differential operator of a function *f*(*t*) defined within the interval [*a*, *t*] is $_{a}D^{q}_{t} f(t)$, with $q \in \text{ R }$.

There exist many definitions for the fractional-order operator (fractional-order integrals for $q<0$ and derivatives for $q>0$) but in this article we will restrict only on the Caputo definition (CD) proposed in 1967 and the Grünwald-Letnikov definition (GLD) proposed in 1867, respectively.

The CD, for $n-1< q <n$, can be written as [20]:

2.1 $$\begin{aligned} _{a}D_{t}^{q}f(t)= \frac{1}{\Gamma (n- q)} \int _{a}^{t} \frac{f^{(n)}(\tau )}{(t-\tau )^{q - n + 1}}d\tau . \end{aligned}$$

In case of real systems with fractional dynamics, where the fractional derivative is used, the Caputo definition can be used because the initial conditions for the fractional differential equations with the Caputo derivatives are in the same form as for ordinary differential equations, i.e. $f^{(n)}(0)=c_n$, $\forall n \in \text{ N }$.

The GLD is given as follows [15, 20]:

2.2 $$\begin{aligned} _{a}D^{q}_{t}f(t)=\lim _{h_s \rightarrow 0}\frac{1}{h_s^{q}} \sum _{j=0}^{\left\lfloor \frac{t-a}{h_s}\right\rfloor }(-1)^j {q \atopwithdelims ()j} f(t-jh_s), \end{aligned}$$

where $\left\lfloor z\right\rfloor$ is the floor function, i.e. the greatest integer smaller than *z*, and

2.3 $$\begin{aligned} {q \atopwithdelims ()j} =\frac{\Gamma (q+1)}{\Gamma (j+1)\,\Gamma (q-j+1)} \end{aligned}$$

are the binomial coefficients with ${q \atopwithdelims ()0} = 1.$ This form of the derivative definition is very helpful for obtaining a numerical solution of the fractional differential equations.

### **Fractional-order systems**

Here, we will consider the following general incommensurate fractional-order nonlinear system represented as [19]:

2.4 $$\begin{aligned} _0D^{q_i}_t x_i (t)= & {} f_i (t, x_1(t), x_2(t), \dots , x_n(t)), \nonumber \\ x_i (0)= & {} c_i, \,\,\, i=1, 2, \dots , n, \end{aligned}$$

where $f_i$ are nonlinear functions and $c_i$ are initial conditions. The vector representation of (2.4) is:

2.5 $$\begin{aligned} D^\mathbf{q } \mathbf{x} = \mathbf{f} (\mathbf{x} ), \end{aligned}$$

where $\mathbf{q} =[q_1, q_2, \dots , q_n]^T$ for $0<q_i<2$, $(i=1, 2, \dots , n)$ and $\mathbf{x} \in \text{ R}^n$.

The equilibrium points of system (2.5) are calculated via solving the following equation

2.6 $$\begin{aligned} \mathbf{f} (\mathbf{x} ) = 0 \end{aligned}$$

and we suppose that $E^{*} = (x_1^{*}, x_2^{*}, \dots , x_n^{*})$ is equilibrium point of system (2.5).

### Numerical solution of initial value problem

It is known that both mentioned definitions, CD and GLD, are equivalent for a wide class of the functions. For numerical calculation of fractional-order derivative we can use the relation (2.7) derived from the GLD (2.2). The expression for the numerical approximation of *q*-th derivative at the points $kh_s,\, (k = 1, 2, 3, \dots )$ has the following general form [20]:

2.7 $$\begin{aligned} _{(k-L_m/h_s)}D^{q}_{t_k} f(t) \approx h_s^{-q} \sum _{j=0}^{k} w_j^{(q)} f(t_{k-j}), \end{aligned}$$

where $L_m$ is the “memory length”, $t_k=kh_s$, $h_s$ is the time step of calculation (definition (2.7) is valid only as $h_s$ tends towards 0 and that the accuracy of the simulation depends on the value of $h_s$) and $w_j^{(q)}\,(j=0, 1, 2, \dots )$ are the binomial coefficients. For their calculation the following expression can be used:

2.8 $$\begin{aligned} w_0^{(q)} = 1, \qquad w_j^{(q)} = \left( 1 - \frac{1+ q}{j}\right) w_{j-1}^{(q)}. \end{aligned}$$

Thus, the numerical solution of the fractional differential equation (initial value problem) of the form

$$\begin{aligned} _0D_t^{q} u(t) = f(t, u(t)), \quad 0 \le t \le T, \end{aligned}$$

can be expressed as follows [19]:

2.9 $$\begin{aligned} u(t_k) = f\left( t_k, u(t_k) \right) h_s^{q} - \sum _{j=1}^{k} w_j^{(q)} u(t_{k-j}). \end{aligned}$$

For the memory term expressed by the sum, a “short memory” principle for various memory length $L_m$ can be used.

An evaluation of the short memory effect and convergence relation of the error between short and long memory were described and proved in [20].

## Fractional-order Lorenz-type systems

The generalized Lorenz system is a 3-dimensional dynamical system with real parameters $a_{11}, a_{12}, a_{21}, a_{22}$, and $\lambda _3$ is given in the following form [1–3]:

3.1 $$\begin{aligned} \left[ \begin{array}{c} \frac{dx(t)}{dt} \\ \frac{dy(t)}{dt} \\ \frac{dz(t)}{dt} \\ \end{array} \right] = \left[ \begin{array}{ccc} a_{11} &{} a_{12} &{} 0 \\ a_{21} &{} a_{22} &{} 0 \\ 0 &{} 0 &{} \lambda _3 \end{array} \right] \left[ \begin{array}{c} x(t) \\ y(t) \\ z(t) \\ \end{array} \right] + x(t) \left[ \begin{array}{ccc} 0 &{} 0 &{} 0 \\ 0 &{} 0 &{} -1 \\ 0 &{} 1 &{} 0 \end{array} \right] \left[ \begin{array}{c} x(t) \\ y(t) \\ z(t) \\ \end{array} \right] , \end{aligned}$$

where

3.2 $$\begin{aligned} a_{11}a_{22}-a_{12}a_{21}<0, \quad a_{11}+a_{22}<0, \quad \lambda _3 <0. \end{aligned}$$

System (3.1) consists of separated linear part and quadratic part and it was a largest possible form that can be considered as a generalized Lorenz system in sense of structural features and the condition given by inequalities in (3.2).

It is easy to see that the above system (3.1) contains as special cases the familiar Lorenz system [11] when $a_{12}a_{21}>0$, the Chen system [7] when $a_{12}a_{21}<0$ and the Lü system [13] when $a_{12}a_{21}=0$.

Following above generalized Lorenz system (3.1) with the conditions (3.2) and taking into account the fractional calculus technique, let us define a new generalized fractional-order Lorenz-type system as follows:

3.3 $$\begin{aligned} \left[ \begin{array}{c} _0D^{q_1}_t x(t) \\ _0D^{q_2}_t y(t) \\ _0D^{q_3}_t z(t) \\ \end{array} \right] = \left[ \begin{array}{ccc} a_{11} &{} a_{12} &{} 0 \\ a_{21} &{} a_{22} &{} 0 \\ 0 &{} 0 &{} a_{33} \end{array} \right] \left[ \begin{array}{c} x(t) \\ y(t) \\ z(t) \\ \end{array} \right] + x(t) \left[ \begin{array}{ccc} 0 &{} 0 &{} 0 \\ 0 &{} 0 &{} -1 \\ 0 &{} 1 &{} 0 \end{array} \right] \left[ \begin{array}{c} x(t) \\ y(t) \\ z(t) \\ \end{array} \right] , \end{aligned}$$

where $q_1$, $q_2$, and $q_3$ are arbitrary derivative orders and $\lambda _3=a_{33}$. In case of $q_1=q_2=q_3=1$ we obtain a classical integer order case defined by expression (3.1). Vector form of (3.3) is given as

$$\begin{aligned} D^{\mathbf{q}} \mathbf{x} = \overline{\mathbf{A}} \mathbf{x} + \mathbf{Q}(\mathbf{x}), \end{aligned}$$

where $\overline{\mathbf{A}}=[ a_{ij}]_{3 \times 3}$ is a real matrix and $\mathbf{Q}(\mathbf{x})$ is quadratic cross-product term.

Relation (3.3) covers the fractional-order version of the Lorenz system [8] for $a_{12}a_{21}>0$, the Chen system [12] for $a_{12}a_{21}<0$, and the Lü system [4] system for $a_{12}a_{21}=0$, respectively.

In order to cover more Lorenz-type systems from a huge family and bridges between them derived from the original Lorenz system during last few decades (see e.g. [16, 23]) as well as their fractional-order versions, we can define even more general fractional-order Lorenz-type system with a class of single quadratic cross-product term $\mathbf{Q} (\mathbf{x} )$, limited to forms $x^2(t)$, *x*(*t*)*y*(*t*) and *x*(*t*)*z*(*t*), which can be written as:

$$\begin{aligned} D^{\mathbf{q}} \mathbf{x} = \overline{\mathbf{A}} \mathbf{x} + \mathbf{Q}(\mathbf{x}) + \mathbf{d}, \end{aligned}$$

where $\mathbf{d}=[d_1, d_2, d_3]^T$ is vector of constants. Extended matrix representation has the form:

3.4 $$\begin{aligned} \left[ \begin{array}{c} _0D^{q_1}_t x(t) \\ _0D^{q_2}_t y(t) \\ _0D^{q_3}_t z(t) \\ \end{array} \right] = \left[ \begin{array}{ccc} a_{11} &{} a_{12} &{} 0 \\ a_{21} &{} a_{22} &{} 0 \\ 0 &{} 0 &{} a_{33} \end{array} \right] \left[ \begin{array}{c} x(t) \\ y(t) \\ z(t) \\ \end{array} \right] + x(t) \left[ \begin{array}{ccc} 0 &{} 0 &{} 0 \\ 0 &{} 0 &{} \tau \\ \mu &{} \delta &{} 0 \end{array} \right] \left[ \begin{array}{c} x(t) \\ y(t) \\ z(t) \\ \end{array} \right] + \left[ \begin{array}{c} d_1 \\ d_2 \\ d_3 \\ \end{array} \right] \,\, \nonumber \\ \end{aligned}$$

or shorted form for $a_{33}=\lambda _3$ as

3.5 $$\begin{aligned} \left[ \begin{array}{c} _0D^{q_1}_t x(t) \\ _0D^{q_2}_t y(t) \\ _0D^{q_3}_t z(t) \\ \end{array} \right] = \left[ \begin{array}{ccc} A &{} 0 \\ 0 &{} \lambda _3 \end{array} \right] \left[ \begin{array}{c} x(t) \\ y(t) \\ z(t) \\ \end{array} \right] + x(t) \left[ \begin{array}{ccc} 0 &{} 0 &{} 0 \\ 0 &{} 0 &{} \tau \\ \mu &{} \delta &{} 0 \end{array} \right] \left[ \begin{array}{c} x(t) \\ y(t) \\ z(t) \\ \end{array} \right] + \left[ \begin{array}{c} d_1 \\ d_2 \\ d_3 \\ \end{array} \right] , \end{aligned}$$

where matrix *A* is given as

3.6 $$\begin{aligned} A= \left[ \begin{array}{cc} a_{11} &{} a_{12} \\ a_{21} &{} a_{22} \\ \end{array} \right] \end{aligned}$$

and the matrix *A* has eigenvalues $\lambda _1, \lambda _2 \in \text{ R }$, where conditions are $\lambda _{1,3}<0$ and $\lambda _2>0$. It is valid only for equilibrium point at the origin $E^{*}=(0;0;0)$. For other equilibria these conditions are not satisfied.

Except the parameters in the matrix *A* and in vector $\mathbf{d}$, we can moderate other parameters $\tau$, $\mu$, and $\delta$ and obtain an additional known Lorenz-type systems, which are presented in this article, as for example, Yang system [25], Liu system [10], Shimizu-Morioka system [22], and Burke-Shaw system [21].

In Table 1 are listed the sets of the model parameters for various chaotic systems presented in this article. It is obvious that by changing the selected system parameters the other type chaotic systems could be obtained as well.

**Table 1:** The values of parameters in (3.5) and (3.6) for various Lorenz-type chaotic systems with $d_1=d_2=0$.

**System**	$a_{11}$	$a_{12}$	$a_{21}$	$a_{22}$	$a_{33}$	$\tau$	$\delta$	$\mu$	$d_3$
Lorenz (3.8)	$-a$	*a*	*c*	$-1$	$-b$	$-1$	1	0	0
Chen (3.11)	$-a$	*a*	$c-a$	*c*	$-b$	$-1$	1	0	0
Lü (3.14)	$-a$	*a*	0	*c*	$-b$	$-1$	1	0	0
Yang (3.17)	$-a$	*a*	*c*	0	$-b$	$-1$	1	0	0
Liu (3.20)	$-a$	*a*	*c*	0	$-b$	$-1$	0	*h*	0
Shimizu-Morioka (3.23)	0	*a*	1	$-c$	$-b$	$-1$	0	1	0
Burke-Shaw (3.26)	$-a$	*a*	0	$-1$	0	$-1$	*g*	0	*d*

In addition, we present the simulation results for seven chaotic systems presented in Table 1 for certain values of the model parameters as well as the fractional orders in the fractional differential equations, respectively. All presented simulations were performed by using relation (2.9) for simulation time $T=100$ s and calculation time step $h_s=0.005$ without using the short memory principle. It means that whole data history was considered for calculation of new value.

We also investigate the stability of these systems in each equilibrium point through eigenvalues [18].

### Lorenz system

The famous Lorenz chaotic system is defined as [11]:

3.7 $$\begin{aligned} \frac{dx(t)}{dt}= & {} a(y(t)-x(t)), \nonumber \\ \frac{dy(t)}{dt}= & {} x(t)(c - z(t)) - y(t), \nonumber \\ \frac{dz(t)}{dt}= & {} x(t)y(t)-b z(t), \end{aligned}$$

where *a* is called the Prandtl number and *c* is called the Rayleigh number. All $a, b, c > 0$, but usually $a = 10$, $b = 8/3$, and $c=28$.

The fractional-order Lorenz system is described as [8]:

3.8 $$\begin{aligned} _0D^{q_1}_t x(t)= & {} a(y(t)-x(t)), \nonumber \\ _0D^{q_2}_t y(t)= & {} x(t)(c- z(t)) - y(t), \nonumber \\ _0D^{q_3}_t z(t)= & {} x(t)y(t)-b z(t), \end{aligned}$$

where $q_1$, $q_2$, and $q_3$ are derivative orders, which could be arbitrary real numbers.

The Lorenz system has three equilibria, where one is obviously in origin $E_1=(0; 0; 0)$ and the additional two for above values of the parameters *a*, *b*, and *c* are: $E_2 = (8.4853;$ 8.4853; 27), and $E_3 = (-8.4853; -8.4853; 27)$. The Jacobian matrix **J** of the Lorenz system at the equilibrium point $E^{*}=(x^{*}, y^{*}, z^{*})$ is given as:

3.9 $$\begin{aligned} \text{ J }=\left[ \begin{array}{ccc} -a &{} a &{} 0 \\ c -z^{*} &{} -1 &{} -x^{*} \\ y^{*} &{} x^{*} &{} -b \\ \end{array} \right] . \end{aligned}$$

For the equilibrium $E_1$ the eigenvalues are $\lambda _1\approx -22.8277$, $\lambda _2 \approx 11.8277$, and $\lambda _3 = -8/3$, and for the equilibria $E_2$ and $E_3$ we get the same eigenvalues $\lambda _1 \approx -13.8546$,  and $\lambda _{2,3} \approx 0.0940 \pm i10.1945$. All three equilibria are unstable.

**Fig. 1:**
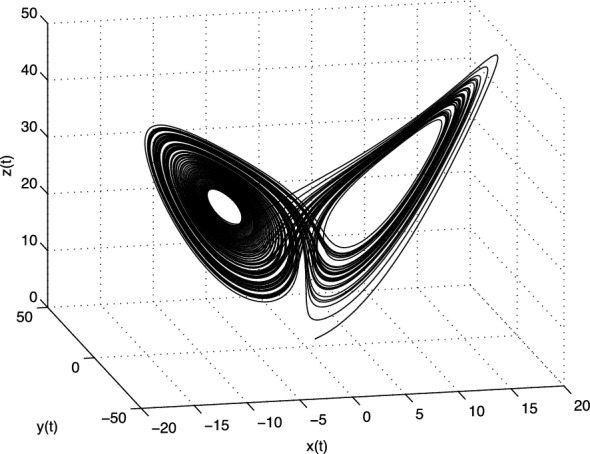
Strange attractor of the fractional-order Lorenz system (3.8) in state space

In Fig. 1 is depicted the simulation result (double-scroll attractor) of the Lorenz system (3.8) for the following parameters: $a = 10,\, b=8/3,\, c=28$, orders $q_1=q_2=q_3=0.993$ and initial conditions $(x(0), y(0), z(0))=(1, 1, 1)$.

### Chen system

In 1999, Chen found another a simple 3-dimensional autonomous system, which is not topologically equivalent to Lorenz system and which has a chaotic attractor too. The Chen chaotic system is described by the following equations [13, 26]:

3.10 $$\begin{aligned} \frac{dx(t)}{dt}= & {} a(y(t)-x(t)), \nonumber \\ \frac{dy(t)}{dt}= & {} (c-a)x(t)-x(t)z(t)+cy(t), \nonumber \\ \frac{dz(t)}{dt}= & {} x(t)y(t)-bz(t), \end{aligned}$$

where $(a,b,c) \in R^3$. When parameters $(a, b, c) = (35, 3, 28)$, the chaotic attractor exists.

The fractional-order Chen system is described as follows [12]:

3.11 $$\begin{aligned} _0D^{q_1}_t x(t)= & {} a(y(t)-x(t)), \nonumber \\ _0D^{q_2}_t y(t)= & {} (c-a)x(t)-x(t)z(t)+cy(t), \nonumber \\ _0D^{q_3}_t z(t)= & {} x(t)y(t)-bz(t), \end{aligned}$$

where $q_1$, $q_2$, and $q_3$ are real derivative orders and where $0<q_1, q_2, q_3 \le 1$.

The equilibrium points of the system with above parameters are: $E_1 = (0; 0; 0)$,  $E_2 = (7.9373; 7.9373; 21)$, and $E_3 = (-7.9373; -7.9373; 21)$. The Jacobian matrix **J** of the Chen system at the equilibrium point $E^{*}=(x^{*}, y^{*}, z^{*}$) is given as

3.12 $$\begin{aligned} \text{ J }=\left[ \begin{array}{ccc} -a &{} a &{} 0 \\ c-a-z^{*} &{} c &{} -x^{*} \\ y^{*} &{} x^{*} &{} -b \\ \end{array} \right] . \end{aligned}$$

For the equilibrium $E_1$ we obtain the eigenvalues $\lambda _1=-30.8359$, $\lambda _2 \approx 23.8359$, and $\lambda _3 = -3$, for the equilibria $E_2$ and $E_3$ we get the same eigenvalues $\lambda _1 \approx -18.4280$,  and $\lambda _{2,3} \approx 4.2140 \pm i14.8846$. All three equilibria are unstable.

**Fig. 2:**
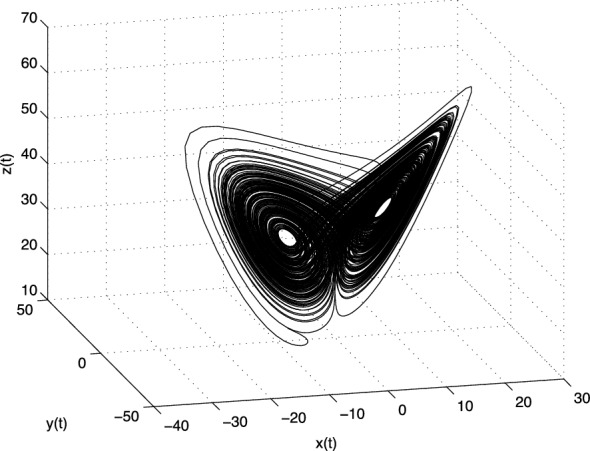
Strange attractor of the fractional-order Chen system (3.11) in state space

In Fig. 2 is depicted the simulation result (double-scroll attractor) of the fractional-order Chen system (3.11) with the parameters $a = 35,\,b = 3,\, c =28$, orders $q_1=q_2=q_3=0.9$, and initial conditions $(x(0), y(0), z(0))=(-9, -5, 14)$.

### Lü system

The so-called Lü system is known as a bridge between the Lorenz system and the Chen system and can be written as [13]:

3.13 $$\begin{aligned} \frac{dx(t)}{dt}= & {} a(y(t)-x(t)), \nonumber \\ \frac{dy(t)}{dt}= & {} -x(t)z(t) + c y(t), \nonumber \\ \frac{dz(t)}{dt}= & {} x(t)y(t) -b z(t), \end{aligned}$$

where the parameters are $a=36$, $b=3$, and *c* varies.

Its fractional-order version is described as follows [4]:

3.14 $$\begin{aligned} _0D^{q_1}_t x(t)= & {} a(y(t)-x(t)), \nonumber \\ _0D^{q_2}_t y(t)= & {} -x(t)z(t) + c y(t), \nonumber \\ _0D^{q_3}_t z(t)= & {} x(t)y(t) -b z(t), \end{aligned}$$

where $0 <q_1, q_2, q_3\le 1$, are arbitrary orders of derivatives, and *a*, *b*, *c* are model parameters.

The Lü system (3.14) has three equilibrium points: $E_1=(0; 0; 0)$, $E_2=(7.7460; 7.7460; 20)$ and $E_3=(-7.7460; -7.7460; 20)$.

The Jacobian matrix $\mathbf{J}$ of the Lü system at the equilibrium point $E^{*}=(x^{*}, y^{*}, z^{*})$ is defined as follows

3.15 $$\begin{aligned} \text{ J }=\left[ \begin{array}{ccc} -a &{} a &{} 0 \\ -z^{*} &{} c &{} -x^{*} \\ y^{*} &{} x^{*} &{} -b \\ \end{array} \right] . \end{aligned}$$

Let us consider the following parameters $a=36,\, b=3,\, c=20$ of the system (3.13). For equilibrium points $E_1$ we obtain the following eigenvalues: $\lambda _1 = -36$, $\lambda _2 = 20$ and $\lambda _3 = -3$. For the equilibria $E_2$ and $E_3$ we have the same eigenvalues $\lambda _1 \approx -22.6516$ and $\lambda _{2,3} \approx 1.8258 \pm i13.6887$. All three equilibria are unstable.

**Fig. 3:**
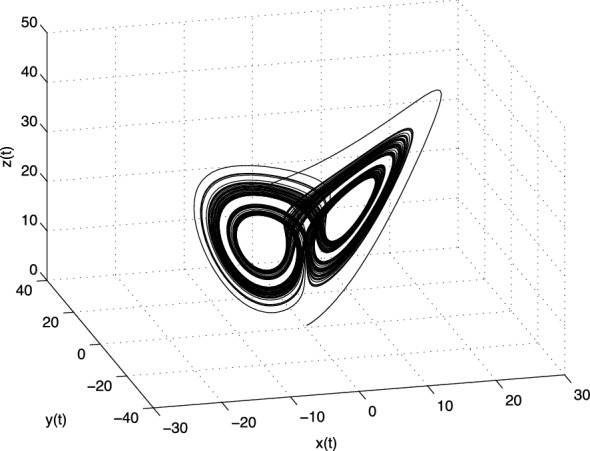
Strange attractor of the fractional-order Lü system (3.14) in state space

In Fig. 3 is depicted the simulation result (double-scroll attractor) of the fractional-order Lü system (3.14) with the parameters $a = 36,\,b = 3,\, c =20$, orders $q_1=0.98$, $q_2=0.99$, $q_3=0.98$, and initial conditions $(x(0), y(0), z(0))=(0.2, 0.5, 0.3)$.

### **Yang system**

The Yang chaotic system is described as follows [25]:

3.16 $$\begin{aligned} \frac{dx(t)}{dt}= & {} a(y(t)-x(t)), \nonumber \\ \frac{dy(t)}{dt}= & {} cx(t)-x(t)z(t), \nonumber \\ \frac{dz(t)}{dt}= & {} x(t)y(t)-bz(t), \end{aligned}$$

where *a*, *b*, *c* are real parameters with $a, b>0$ and $c \in \text{ R }$. When $a=10$, $b=8/3$, and $c=16$, this system is chaotic. The algebraical form of the chaotic attractor is very similar to the Lorenz-type systems but they are different and, in fact, nonequivalent in topological structures.

Its fractional-order version can be described as follows:

3.17 $$\begin{aligned} _0D^{q_1}_t x(t)= & {} a(y(t)-x(t)), \nonumber \\ _0D^{q_2}_t y(t)= & {} cx(t)-x(t)z(t), \nonumber \\ _0D^{q_3}_t z(t)= & {} x(t)y(t)-bz(t), \end{aligned}$$

where $q_1$, $q_2$, and $q_3$ are derivative orders, which could be arbitrary real numbers.

The Yang system (3.17) with above parameters has three equilibrium points: $E_1=(0; 0; 0)$, $E_2=(6.5320; 6.5320; 16)$ and $E_3=(-6.5320; -6.5320; 16)$.

The Jacobian matrix $\mathbf{J}$ of the Yang system for the equilibrium point $E^{*}=(x^{*}, y^{*}, z^{*})$ is defined as follows

3.18 $$\begin{aligned} \text{ J }=\left[ \begin{array}{ccc} -a &{} a &{} 0 \\ c-z^{*} &{} 0 &{} -x^{*} \\ y^{*} &{} x^{*} &{} -b \\ \end{array} \right] . \end{aligned}$$

For equilibrium points $E_1$ we obtain the following eigenvalues: $\lambda _1\approx -18.6015$, $\lambda _2 \approx 8.6015$ and $\lambda _3 = -8/3$. For the equilibria $E_2$ and $E_3$ we have the same eigenvalues $\lambda _1 \approx -12.5570$ and $\lambda _{2,3} \approx -0.0548 \pm i8.2434$. The equilibrium $E_1$ is unstable and the equilibria $E_2$ and $E_3$ are stable.

**Fig. 4:**
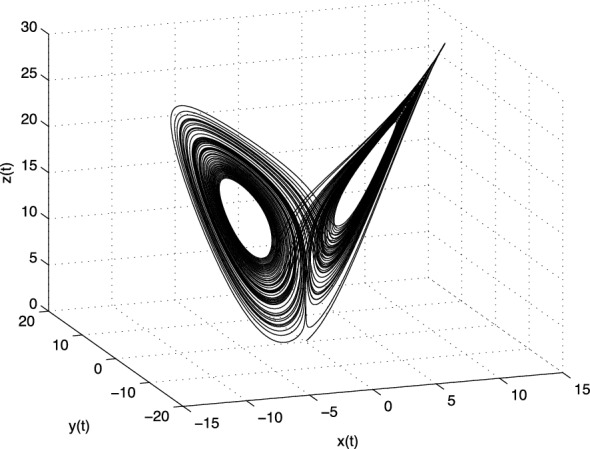
Strange attractor of the fractional-order Yang system (3.17) in state space

In Fig. 4 is depicted the simulation result (double scroll-attractor) of the fractional-order Yang system (3.17) with parameters $a = 10,\,b = 8/3,\, c =16$, orders $q_1=q_2=q_3=0.99$, and initial conditions $(x(0), y(0), z(0))=(0.1, 0.1, 0.1)$.

### **Liu system**

Another system similar to the Lorenz chaotic system was proposed in [10]:

3.19 $$\begin{aligned} \frac{dx(t)}{dt}= & {} a(y(t)-x(t)), \nonumber \\ \frac{dy(t)}{dt}= & {} cx(t)- k x(t) z(t), \nonumber \\ \frac{dz(t)}{dt}= & {} -b z(t) + h x^2(t). \end{aligned}$$

The system exhibits chaotic behaviour for parameters $a = 10$, $b=2.5$, $c = 40$, $k=1$, and $h=4$.

Following the previously published fractional-order version of the Lorenz family systems, let us define a fractional-order version of system described by (3.19), which has the following form:

3.20 $$\begin{aligned} _0D^{q_1}_t x(t)= & {} a(y(t)-x(t)), \nonumber \\ _0D^{q_2}_t y(t)= & {} cx(t)- k x(t) z(t), \nonumber \\ _0D^{q_3}_t z(t)= & {} -b z(t) + h x^2(t), \end{aligned}$$

where $q_1$, $q_2$, $q_3$ are derivative orders, which could be arbitrary real numbers.

The Liu system (3.20) with above parameters has three equilibrium points: $E_1=(0; 0; 0)$, $E_2=(5; 5; 40)$ and $E_3=(-5; -5; 40)$.

The Jacobian matrix $\mathbf{J}$ of the Liu system for the equilibrium point $E^{*}=(x^{*}, y^{*}, z^{*})$ is defined as follows

3.21 $$\begin{aligned} \text{ J }=\left[ \begin{array}{ccc} -a &{} a &{} 0 \\ c-kz^{*} &{} 0 &{} -kx^{*} \\ 2hx^{*} &{} 0 &{} -b \\ \end{array} \right] . \end{aligned}$$

For equilibrium points $E_1$ we obtain the following eigenvalues: $\lambda _1 \approx -25.6155$, $\lambda _2 \approx 15.6155$ and $\lambda _3 = -2.5$. For the equilibria $E_2$ and $E_3$ we get the same eigenvalues $\lambda _1 \approx -17.5614$ and $\lambda _{2,3} \approx 2.5307 \pm i10.3673$. All three equilibria are unstable.

**Fig. 5:**
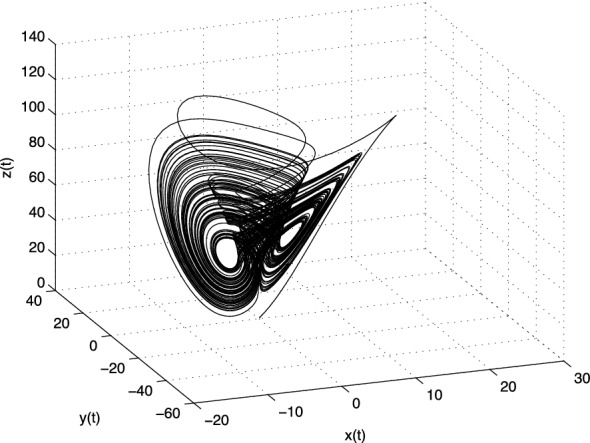
Strange attractor of the fractional-order Liu system (3.20) in state space

In Fig. 5 is depicted the simulation result (double scroll-attractor) of the fractional-order Liu system (3.20) with parameters $a = 10,\,b = 2.5,\, c =40,\,k=1,\,h=4$, orders $q_1=q_2=q_3=0.95$, and initial conditions (*x*(0), *y*(0), $z(0))=(0.2, 0, 0.5)$.

Obviously, the strange attractor of the fractional-order Liu system (3.20), shown in Fig. 5, is slightly different to the Lorenz attractor depicted in Fig. 1.

### **Shimizu-Morioka system**

A simple model, the solution of which shows a behaviour as in the Lorenz model for high Rayleigh numbers was proposed in [22]:

3.22 $$\begin{aligned} \frac{dx(t)}{dt}= & {} a y(t), \nonumber \\[4pt] \frac{dy(t)}{dt}= & {} x(t)(1 - z(t)) - c y(t), \nonumber \\[4pt] \frac{dz(t)}{dt}= & {} -bz(t) + x^2(t), \end{aligned}$$

where we added an additional parameter *a* in the first equation.

Its fractional-order version was suggested in [27] and has the form:

3.23 $$\begin{aligned} _0D^{q_1}_t x(t)= & {} a y(t), \nonumber \\[4pt] _0D^{q_2}_t y(t)= & {} x(t)(1 - z(t)) - c y(t), \nonumber \\[4pt] _0D^{q_3}_t z(t)= & {} -b z(t) + x^2(t), \end{aligned}$$

where $q_1$, $q_2$, $q_3$ are derivative orders, which could be arbitrary real numbers.

The Shimizu-Morioka system (3.23) with above parameters has three equilibrium points: $E_1=(0; 0; 0)$, $E_2=$ (0.61237;  0; 1) and $E_3=(-0.61237; 0; 1)$.

The Jacobian matrix $\mathbf{J}$ of the Shimizu-Morioka system for the equilibrium point $E^{*}=(x^{*}, y^{*}, z^{*})$ is defined as follows

3.24 $$\begin{aligned} \text{ J }=\left[ \begin{array}{ccc} 0 &{} a &{} 0 \\ 1-z^{*} &{} -c &{} -x^{*} \\ 2x^{*} &{} 0 &{} -b \\ \end{array} \right] . \end{aligned}$$

For equilibrium points $E_1$ we obtain the following eigenvalues: $\lambda _1 \approx -1.4839$, $\lambda _2 \approx 0.6739$ and $\lambda _3 = -0.375$. For the equilibria $E_2$ and $E_3$ we have the same eigenvalues $\lambda _1 \approx -1.3650$ and $\lambda _{2,3} \approx 0.0900 \pm i0.7358$. All three equilibria are unstable.

**Fig. 6:**
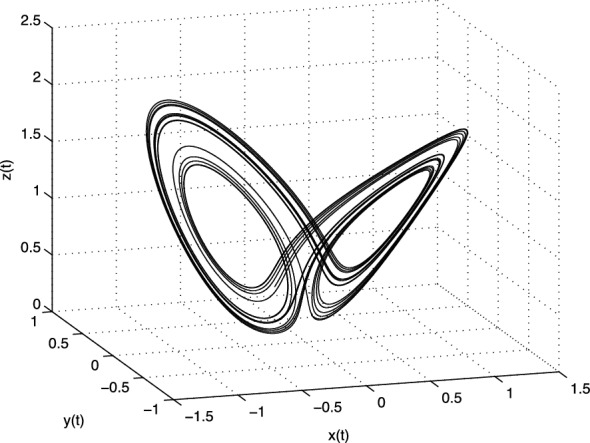
Strange attractor of the fractional-order Shimizu-Morioka system (3.23) in state space

In Fig. 6 is depicted the simulation result (double scroll-attractor) of the fractional-order Shimizu-Morioka system (3.23) with parameters $a=1$, $b = 0.375$, $c =0.81$, orders $q_1=0.95$, $q_2=0.97$, $q_3=0.99$, and initial conditions (*x*(0), *y*(0),  $z(0))=(0.1, 0.1, 0.2)$.

It can be proved that there is a large open region in the (*b*, *c*)-plane where the Shimizu-Morioka system has a strange attractor very similar to the classical attractor of the Lorenz model.

### **Burke-Shaw system**

The Burke-Shaw system was derived by Burke and Shaw from the Lorenz system [21]. This system has similar algebraic structure to the Lorenz system but is topologically nonequivalent to the generalized Lorenz-type system and can be expressed as follows:

3.25 $$\begin{aligned} \frac{dx(t)}{dt}= & {} -a (x(t) + y(t)), \nonumber \\ \frac{dy(t)}{dt}= & {} -k x(t) z(t) -y(t), \nonumber \\ \frac{dz(t)}{dt}= & {} g x(t)y(t) + d, \end{aligned}$$

where for parameters $a=k=g=10$ and $d=13$ or $d=4.272$ chaos is observed.

Its fractional-order version can be expressed as [14]:

3.26 $$\begin{aligned} _0D^{q_1}_t x(t)= & {} -a (x(t) + y(t)), \nonumber \\ _0D^{q_2}_t y(t)= & {} -k x(t) z(t) -y(t), \nonumber \\ _0D^{q_3}_t z(t)= & {} g x(t)y(t) + d, \end{aligned}$$

where $q_1$, $q_2$, $q_3$ are derivative orders, which could be arbitrary real numbers.

The Burke-Shaw system (3.26) with above parameters has two equilibrium points: $E_1=(1.1402; -1.1402; 0.1)$ and $E_2=(-1.1402; 1.1402; 0.1)$. Obviously, due to parameter *d* in the model there is no equilibrium point at the origin.

The Jacobian matrix $\mathbf{J}$ of the Burke-Shaw system for the equilibrium point $E^{*}=(x^{*}, y^{*}, z^{*})$ is defined as follows

3.27 $$\begin{aligned} \text{ J }=\left[ \begin{array}{ccc} -a &{} -a &{} 0 \\ -kz^{*} &{} -1 &{} -kx^{*} \\ gy^{*} &{} gx{*} &{} 0 \\ \end{array} \right] . \end{aligned}$$

For the equilibria $E_1$ and $E_2$ we obtain the same eigenvalues $\lambda _1 \approx -14.4527$ and $\lambda _{2,3} \approx 1.7263 \pm i13.3013$. Both equilibria are unstable.

**Fig. 7:**
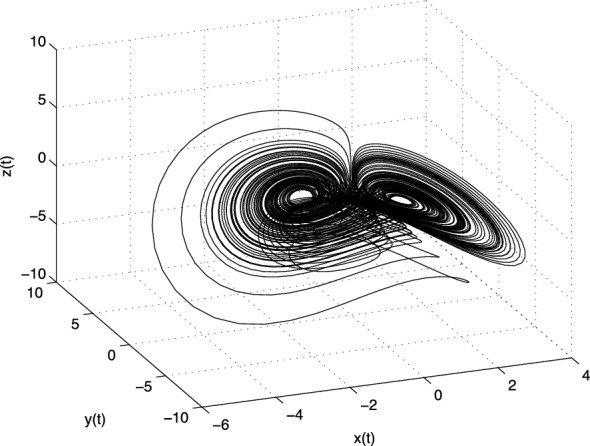
Strange attractor of the fractional-order Burke-Shaw system (3.26) in state space

In Fig. 7 is depicted the simulation result (double scroll-attractor) of the fractional-order Burke-Shaw system (3.26) with parameters $a=k=g=10,\,d=13$, orders $q_1=0.95$,  $q_2=0.97$,   $q_3=0.99$, and initial conditions (*x*(0), *y*(0), *z*(0)) = (0.1, 0.1, 0.1).

It should be noted, that various Lorenz-type systems are interconnected and they could be topologically equivalent or not. The Burke-Shaw system is topologically equivalent to the Lorenz system under the transformation [6].

## Conclusions

In this survey paper we tried to classified a wide scale of the Lorenz-type systems, which were investigated during last years by many authors. Those authors detailed investigated the Lorenz-type chaotic systems and their characteristics as Lyapunov exponents, fractal dimensions, stability, attractors, biffurcation diagrams as well Poincaré maps, etc. However, we did not repeated this investigation again. Here we considered the fractional-order modifications of these type systems by illustrative examples. Moreover, for the first time a new general form of the fractional-order Lorenz-type system (3.5) was presented as well. New general form defined in this paper covers not only known systems and bridges between them but even conjugate systems [24] also know as hyperbolic system [3], when parameter $\tau =1$ in (3.5). Some other Lorenz-type systems, which are not considered in this article are also particular cases of the general form (3.5), defined in this paper, as for example, Sprott systems (a few of 19 examples in [23]), Li system [9], Pehlivan-Uyaroǧlu system [17], model for El-Niño weather phenomenon [5] as well as a whole zoo of systems from the Lorenz-type family (several of 150 examples described in [16]).

There are also higher dimension Lorenz model as well as other Lorenz-type systems with a different quadratic form. However, in this brief survey presented in this article we did not consider them. It is an idea for further research.
